# Efficacies of Conventional Antifungals and Complementary and Alternative Medicine as Single or Combination Therapies Against *Candida* Biofilms in Recurrent Vaginal Candidiasis: An In Vitro Study

**DOI:** 10.3390/jof12060415

**Published:** 2026-06-08

**Authors:** Yihong Pan, Liumei Ye, Lanqian Chen, Lauren Hermann, Panpan Jin, Yingying Cai, Yali Cheng, Weidan Zhang, Cathy J Watson, David McGiffin, Qiong Luo, Xueqiong Zhu, Yue Qu

**Affiliations:** 1 The Consortium for Infection and Innovation (CII), The First Affiliated Hospital of Wenzhou Medical University, Wenzhou 325000, China; 2 Department of Obstetrics and Gynaecology, Taizhou Hospital of Zhejiang Province Affiliated to Wenzhou Medical University, Taizhou 318000, China; 3 Department of Pathology, Taizhou Hospital of Zhejiang Province Affiliated to Wenzhou Medical University, Taizhou 318000, China; 4 Department of Microbiology, Biomedical Discovery Institute, Monash University, Clayton 3800, Australia; 5 Department of Laboratory Medicine, Taizhou Hospital of Zhejiang Province Affiliated to Wenzhou Medical University, Taizhou 318000, China; 6 School of Population and Global Health, University of Melbourne, Carlton 3053, Australia; 7 Department of Cardiothoracic Surgery, The Alfred and Monash University, Melbourne 3004, Australia; 8 Department of Obstetrics, Women’s Hospital, Zhejiang University School of Medicine, Hangzhou 310000, China; luoq@zju.edu.cn; 9 Department of Obstetrics and Gynaecology, The 2nd Affiliated Hospital of Wenzhou Medical University, Wenzhou 325000, China

**Keywords:** RVVC, conventional antifungals, complementary and alternative medicine, drug combinations, synergy, anti-biofilms

## Abstract

**Objectives:** Recurrent vulvovaginal candidiasis (RVVC) is a difficult-to-treat infection, most likely due to the growth of *Candida* biofilms on the human vaginal epithelium. We assessed in vitro efficacy of conventional antifungals and complementary and alternative medicine (CAM) used in clinical settings, and sought for *Candida* biofilm-effective single or combination therapies. **Methods:** Standard broth microdilution assay and XTT (2,3-Bis-(2-Methoxy-4-Nitro-5-Sulfophenyl)-2H-Tetrazolium-5-Carboxanilide) assay were used for antifungal and anti-biofilm efficacies of three conventional antifungals, and selected CAM including boric acid, povidone-iodine, and allicin (garlic extract), against *Candida* clinical isolates grown at neutral and acidic pHs respectively. Fractional inhibitory concentration (FIC) indices were assessed to evaluate interactions between fluconazole and different CAM. Viable count-based cell enumeration and confocal laser scanning microscopy (CLSM) were performed to confirm the efficacy of single or combination therapies against *Candida* biofilms. **Results:** All selected conventional antifungals and CAM showed efficacies against planktonic *Candida* cells. Acidic vaginal microenvironments provided agent-specific protection to *Candida* cells against conventional antifungals and the CAM. Synergistic or additive interactions were observed between fluconazole at serum achievable concentrations and povidone-iodide at topically achievable concentrations against all tested *Candida* strains. Most antifungal agents except caspofungin had very limited activities against *Candida* biofilms. Combining fluconazole at 8 mg/L with povidone-iodine at 2048 mg/L effectively killed *Candida* biofilms in an acidic vaginal microenvironment to a level that is comparable to that of caspofungin. **Conclusions:** We provided robust in vitro evidence supporting the combinational use of oral fluconazole and topical CAM povidone-iodine against *Candida* biofilms in managing RVVC.

## 1. Introduction

Vulvovaginal candidiasis (VVC) is a *Candida* infection of the lower reproductive tract that affects approximately 70–75% of women of reproductive age, with 5–8% of patients experiencing three or more episodes within a 12-month period, a difficult-to-treat medical condition known as recurrent VVC or RVVC [1,2,3]. Conventional antifungal drugs such as fluconazole are recommended by the Centers for Disease Control and Prevention (CDC) for the management of VVC or RVVC, typically based on susceptibility testing of planktonically grown yeast cells [3,4,5,6]. Although CDC-recommended conventional drugs can alleviate clinical symptoms of RVVC, they often fail to eliminate the causative pathogen or to completely cure the infection [7,8,9,10].

*Candida* species are among common opportunistic pathogens that can adopt a major pathogenicity strategy, biofilm formation, to initiate and maintain infections [11,12]. Biofilms are three-dimensional microbial communities embedded within a matrix of host-derived components and self-produced extracellular polymeric substances (EPSs) [13]. Within this architecture, biofilm cells exhibit pronounced physiological heterogeneity, altered transcriptional and translational profiles and enhanced survival capacity [14]. A hallmark feature of biofilms is their marked tolerance to antimicrobial agents, often exceeding that of their planktonic counterparts by several orders of magnitude [15,16,17,18]. We recently established experimental and clinical evidence of biofilm formation of *Candida* spp. on the vaginal epithelium, supporting an important role of *Candida* biofilms in the pathogenesis of RVVC [19,20]. We also proposed that an ideal antifungal drug for RVVC should be highly effective against *Candida* biofilms grown on the vaginal epithelium [19,20]. In the widely used treatment guideline for VVC, the CDC recommends conventional antifungals such as topical clotrimazole, or oral fluconazole for uncomplicated cases, and prolonged oral fluconazole for RVVC [3]. Among conventional antifungals, echinocandins such as caspofungin, micafungin, and anidulafungin have been shown to be superior to fluconazole and amphotericin B in killing *Candida* biofilms in vitro; however, echinocandins require intravenous administration and are associated with very high cost [21,22,23,24].

Due to the limited availability and suboptimal long-term effectiveness of conventional antifungals for RVVC, complementary and alternative medicine (CAM) such as allicin (a compound of garlic), povidone-iodine (Betadine) and boric acid, has been experimentally and clinically evaluated, demonstrating controversial or sometimes promising clinical effectiveness [25,26,27,28,29]. Boric acid is recommended by the CDC for non-*albicans* RVVC cases that do not respond to either prolonged fluconazole or a non-fluconazole azole regimen [3]. Beyond their notable adverse effects in the vagina, including burning sensation, watery discharge, and erythema, the clinical efficacy of CAM has been reported to be modest and inconsistent [25,30,31]. There is very limited information in the literature regarding the efficacy of CAM therapies against *Candida* biofilms, one of the major contributors to the pathogenesis of RVVC [27]. It also remained to be clarified whether CAM should be used as complementary or alternative therapies for the CDC-recommended oral fluconazole treatment.

This study aims to assess the in vitro efficacy of conventional antifungals and three CAMs that have been approved or experimentally used in clinical settings against *Candida* causing RVVC, and to seek for biofilm-effective monotherapies, or combinational treatments with synergetic interactions between single components.

## 2. Materials and Methods

### 2.1. Strains and Growth Conditions

Five *Candida* strains, including a laboratory reference strain *C. albicans* SC5314 and four VVC/RVVC clinical isolates, were used in this study. *C. albicans* SC5314 is a well-recognized biofilm producer; this strain or its derivative strains have been widely used in biofilm studies [20,32,33]. *C. albicans* VVC4 and VVC2 were clinical isolates from an RVVC patient and a VVC patient visiting the First Affiliated Hospital of Wenzhou Medical University respectively [20]. *C. albicans* QY3 and *C. glabrata* QY4 were sampled from the vaginal epithelium of RVVC patients at the Taizhou Enze Hospital of Wenzhou Medical University [19]. All strains were stored at −80 °C in 15% (*v*/*v*) glycerol and streaked onto YPD plates (1% yeast extract, 2% peptone, 2% glucose) as working stocks.

### 2.2. Antifungal Agents

Three conventional antifungals and three CAM agents that have been clinically or experimentally used to treat RVVC were selected. The conventional antifungals included fluconazole (CAS 86386-73-4, Macklin Biochemical, Shanghai, China), clotrimazole (CAS 23593-75-1, Macklin Biotechnology, Shanghai, China) and caspofungin as a representative of the echinocandin class (CAS 179463-17-3, Macklin Biochemical, Shanghai, China), and the three CAM agents were Allicin (Garlicin, Nature’s way, Green Bay, WI, USA), povidone-iodine (CAS 25655-41-8, Macklin Biochemical, Shanghai, China), and boric acid (CAS 10043-35-3, Macklin Biochemical, Shanghai, China). Stock solutions were prepared in sterile distilled H_2_O to 6400 mg/L for fluconazole and 8196 mg/L for all other drugs and diluted into RPMI-1640 as working solutions.

### 2.3. Antifungal Susceptibility Tests for Planktonic Cultures

Minimum inhibitory concentrations (MICs) of conventional antifungals and CAM were determined using the reference method for broth dilution antifungal susceptibility testing of yeasts (CLSI guideline M27-A3). The inoculated microplates were incubated at 35 °C for 24 h (caspofungin and three CAMs) or 48 h (clotrimazole and fluconazole). A microplate reader (Tecan) was used to assess the inhibition of fungal growth by different agents. The MIC was defined as the lowest concentration that inhibited ≥50% of fungal growth for clotrimazole and fluconazole, or with no visible growth for all other agents. The minimal fungicidal concentration (MFC) assay was also carried out for microwells without visible fungal growths after MIC determination. A 20 µL aliquot was plated on YPD agar and incubated at 35 °C for 48 h prior to colony-forming unit (CFU) enumeration. The MFC was the lowest concentration causing at least 2-log reduction in the initial inoculum [21]. RPMI-MOPS of pH = 7.2 or pH = 4.2 were prepared respectively and used as growth media; the former was recommended by the CLSI guideline and the latter was adopted to reflect the acidic vaginal microenvironment of VVC/RVVC patients.

### 2.4. Antifungal Interaction Testing

A checkerboard method was used to assess drug interactions between the CDC-recommended fluconazole and three CAM agents [34]. The MICs for single components and the combination-derived MICs were determined to calculate the fractional inhibitory concentration (FIC) index. The drug combination was categorized as synergistic with an FIC ≤ 0.5, additive with an FIC between 0.5 and 1.0, indifferent with an FIC between 1 and 4, and antagonistic when the FIC was >4. The drug interactions were examined under a neutral pH (7.2) and an acidic pH (4.2) respectively. MIC_80_, the minimum concentration causing 80% reduction in fungal viability compared to the drug-free control, was adopted for fluconazole in the checkerboard assay, following the published method by Liu et al. (2017) [35].

### 2.5. Antifungal Susceptibility Tests for Biofilms

To obtain comparable data on the efficacy of conventional antifungals and CAM against planktonic cultures and biofilms, the lowest concentration of agents that inhibited ≥50% of biofilm growth was determined and reported as BMIC_50_.

To grow in vitro biofilms, overnight *Candida* cultures were grown in YPD broth and diluted into RPMI-1640 of pH = 7.2 to a cell density of ~1 × 10^6^ colony-forming units (CFUs) per mL. One hundred microlitre aliquots of diluted fungal suspensions were pipetted into wells of a 96-well microplate and incubated for 24 h at 37 °C with gentle agitation (75 rpm). After incubation, the suspensions were aspirated and the microwells were rinsed with phosphate-buffered saline (PBS) to remove non-adherent cells. Two hundred microlitre aliquots of 2-fold serial dilutions of antifungal agents or their combinations were added into each well. The treatment was consistent with that for planktonic cultures and lasted for 24 or 48 h for *Candida* biofilms. The BMIC_50_ was determined by measuring the OD_492_ of drug-treated biofilms using the XTT assay, as previously described [32]. More accurate CFU-based biofilm cell enumeration was carried out to confirm the efficacy of antifungals [32]. To do that, biofilms were dissociated from the surface of microwells after treatment with PBS, by scraping the surface with sterile cotton swabs. The suspension and cotton swab were transferred into an Eppendorf tube containing 1 mL of sterile PBS, vortexed for 30 s at maximum speed four times and sonicated in a sonication bath at 42 kHz for 10 min. The cell numbers of fungi were determined after plating serial dilutions onto YPD plates followed by overnight growth.

### 2.6. Confocal Laser Scanning Microscopy

Fungal biofilm cultures were set up on tissue culture-treated coverslips in 24-well microplates. The microwell containing the coverslip was inoculated with 1 mL of *Candida* suspension prepared in RPMI-1640 (∼10^6^ cfu/mL), followed by overnight incubation at 35 °C and washing with 0.9% saline to remove planktonic cells. For biofilm morphology and structure, fungal cells grown on coverslips were stained with ConA (1 mg/mL, Sigma-Aldrich, Shanghai, China) for 45 min, Calcofluor white (1 g/L, Sigma-Aldrich, Shanghai, China) for 1 min and then examined with a confocal laser scanning microscope (Leica SP8) [36]. For cell viability after antifungal treatment, biofilms were stained with the BacLight Live/Dead Viability Kit (L7007, Invitrogen, containing 3.35 μM SYTO-9 and 20 μM propidium iodide) at 35 °C for 30 min in the dark, washed twice with saline and viewed using the Leica SP8 with the ×40 objective [32]. Three-dimensional biofilm images were reconstructed with the LAS X software (V 3.10).

### 2.7. Statistical Analysis

To analyze differences in the biofilm cell growth or survival after antifungal treatments, one-way ANOVA tests or non-parametric assays were performed with Minitab 16 for Windows^®^ using a significance level of 0.05 (*p* value).

## 3. Results

### 3.1. In Vitro Biofilm Formation of Candida Strains Selected for This Study

**Figure 1:**
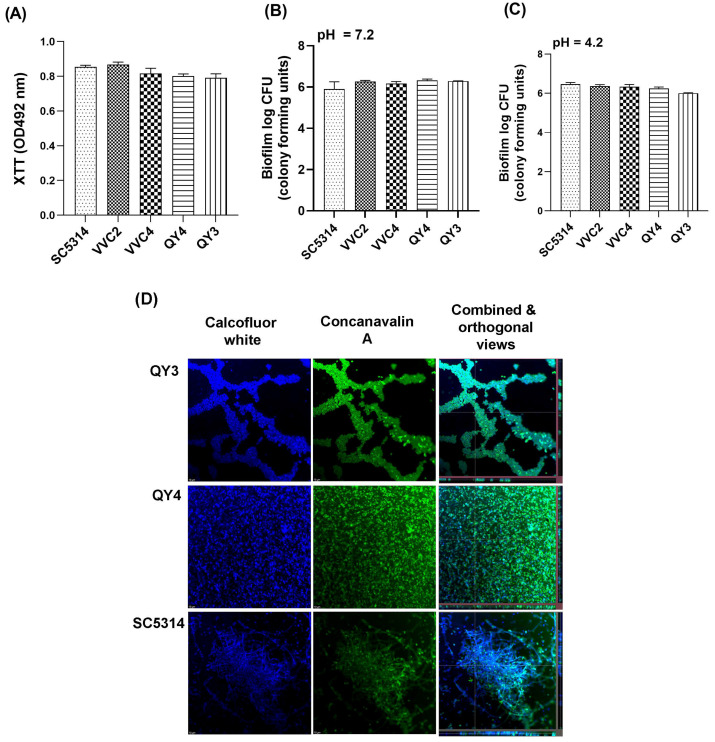
In vitro biofilm formation by *Candida* vaginal isolates: (**A**) Semi-quantitative XTT assays. (**B**) Viable count-based biofilm assay at pH = 7.2. (**C**) Viable count-based biofilm assay at pH = 4.2. All three assays show all tested clinical isolates produced in vitro biofilms; no difference is found in biofilm quantities between the three RVVC isolates and the VVC strain, or clinical isolates and SC5314. (**D**) Confocal laser scanning microscopy in combination with calcofluor white (staining fungal cells) and ConA [staining extracellular matrix (ECM)] show that vaginal isolates formed yeast-dominated biofilms, morphologically distinct from biofilms formed by *C. albicans* SC5314, which is characterized by a basal layer of yeast cells covered by multiple layers of hyphal cells. These XTT and viable count-based biofilm assays are carried out in three biological repeats in technical triplicate.

Semi-quantitative XTT assays showed all four selected clinical isolates and the laboratory reference strain SC5314 produced in vitro biofilms when grown in RPMI-1640 at a neutral pH of 7.2 (Figure 1A); no difference was found in biofilm quantities between the clinical isolates, or between clinical isolates and SC5314. This was further confirmed by the more accurate cell enumeration-based CFU analysis of biofilms grown at pH 7.2 or the acidic pH of 4.2 (Figure 1B,C). Structural analysis of biofilms using CLSM in combination with ConA and calcofluor white showed that the representative clinical isolates QY3 and QY4 formed yeast-dominating biofilms embedded in EPS (Figure 1D), morphologically similar to clinical biofilms observed on infected vaginal tissues [19]. The *C. albicans* reference strain SC5314 formed a contrasting biofilm under the same conditions, with numerous layers of filamentous cells projecting from a basal layer of yeast cells (Figure 1D).

### 3.2. Antifungal Susceptibilities of Conventional Antifungals and CAM Agents Against Candida Planktonic Cells

All four clinical isolates remained susceptible to all three conventional antifungals, with MICs and MFCs ranging from 0.125 to 1 mg/L and 0.25 to 1 mg/L respectively for fluconazole, both 0.125 mg/L for caspofungin, and both 0.25–1 mg/L for clotrimazole when tested using the standard CLSI-recommended broth microdilution method (pH = 7.2) (Table 1). Three CAM agents also demonstrated activities against *Candida* vaginal isolates, with MICs and MFCs ranging from 256 to 2048 mg/L, exponentially higher than that of conventional antifungals (Table 1).

We also assessed antifungal susceptibilities of conventional and CAM drugs at pH 4.2, an acidic condition that reflects the vaginal microenvironment of VVC/RVVC patients. Lower pHs had an agent- and strain-dependent impact on the activity of antifungals or CAM agents, increasing the MIC and MFCs of clotrimazole by 2–16 folds in 5/5 strains, that of boric acid by ≥2 folds in 5/5 strains, and reducing MICs and MFCs of povidone-iodide by ≥2 folds in 4/5 strains (Table 1).

### 3.3. Combined Effects of Conventional and CAM Agents Against Candida Planktonic Cultures

We further examined interactions between the CDC-recommended fluconazole and three CAMs, using the checkerboard method. Fluconazole and povidone-iodine demonstrated synergic or additive interactions against all vaginal isolates at both pH values, with the minimum FIC index ranging from 0.31 to 0.56, indicating synergism or a trend toward synergism (Table 2). Mostly additive or indifferent interactions were observed when boric acid was combined with fluconazole under neutral or acidic conditions. Different interactions were found when Allicin was added to fluconazole and the effects appeared to be strain-specific. These results suggested that combining fluconazole and povidone-iodine may be more effective in killing *Candida* cells than single conventional antifungals or selected CAM therapies.

### 3.4. Antifungal Susceptibilities of Conventional or CAM Antifungals Against Biofilms Formed by Candida Vaginal Isolates

XTT assay was used to screen the anti-biofilm efficacy of conventional antifungals and CAM agents, either as single or combination therapies. Among the conventional antifungals and CAM agents, caspofungin showed the greatest anti-biofilm efficacy, with the lowest BMIC_50_ ranging from 4 to 32 mg/L (Table 3). All other agents failed to achieve 50% of biofilm inhibition at very high concentrations (32 to >128 mg/L for fluconazole and clotrimazole, and 512 to >4096 mg/L for boric acid, allicin and povidone-iodine, see Table 3).

Combining fluconazole at 8 mg/L, a serum achievable concentration [37], with povidone-iodine but not the other CAM in the XXT screening assay clearly showed synergistic or additive effects against *Candida* biofilms, allowing povidone-iodine to achieve a 50% biofilm reduction at a concentration that was at least 2-fold lower in 5/5 strains (Table 3). This assay was only carried out at pH = 7.2, due to the sensitivity issue of XTT assay at a lower pH, as found in our preliminary study.

### 3.5. Combining Fluconazole and Povidone-Iodine Successfully Killed Candida Biofilms and Outperformed Single Therapies in an Acidic Environment

**Figure 2:**
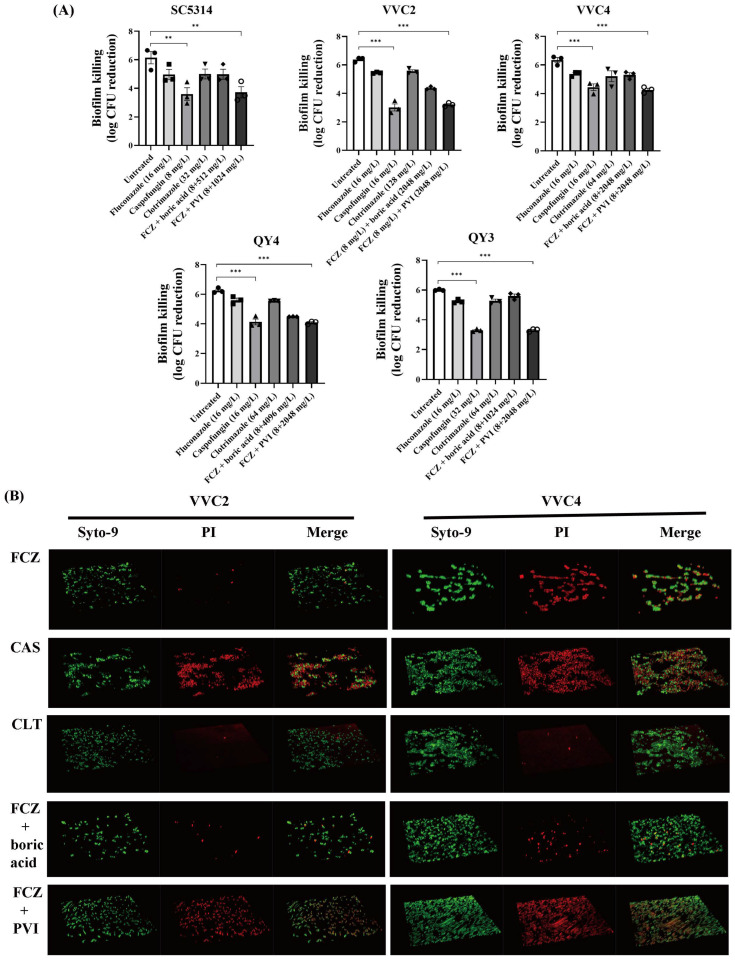
Biofilm killing by conventional antifungals and CAM agents as single agents or in combination: (**A**) Viable count-based biofilm killing assay. Serum-achievable concentrations (for fluconazole and caspofungin that are administered systemically) or BMIC_50_ (for clotrimazole and CAM agents that are administered topically) are selected for each conventional antifungal or CAM. (**B**) Confocal laser scanning microscopy analysis of biofilms formed by two representative *Candida* strains (VVC2 and VVC4) after antifungal treatment. Concentrations of antifungal agents refer to those in Figure 2A. CLSM three-dimensional reconstructions show the staining pattern for live cells (SYTO-9, green) and dead cells (propidium iodide, red). FCZ, fluconazole; CAS, caspofungin; CLT, clotrimazole; and PVI, povidone-iodine. The experiments are performed twice and representative images are shown. **, *p* < 0.01, ***, *p* < 0.001.

We further confirmed the anti-biofilm efficacy of conventional antifungals and CAM, as single or combinational therapies at the vaginal pH of 4.2, using the quantitative biofilm CFU assay and a qualitative CLSM. Antifungals at serum achievable concentrations or BMIC_50_ by topical application were used to treat established fungal biofilms (see Figure 2A). Caspofungin at serum achievable concentrations showed the greatest anti-*Candida*-biofilm activities, killing mature *Candida* biofilms by 2.38 ± 0.84 log units. In contrast, fluconazole at 16 mg/L, clotrimazole at 32–128 mg/L only killed *Candida* biofilms by 0.89 ± 0.40 and 1.05 ± 0.61 log units respectively. Combining fluconazole at 8 mg/L with boric acid showed greater anti-biofilm activities against 2/5 strains (VVC2 and QY4) and similar efficacy for the remaining 3/5 strains, in comparison with fluconazole alone. Fluconazole at 8 mg/L in combination with povidone-iodine at 1024–2048 mg/L demonstrated excellent biofilm killing against all tested strains (2.30 ± 0.81 log reduction), comparable to that of caspofungin and higher than fluconazole or clotrimazole alone, or the fluconazole and boric acid combination (*p* < 0.05). Qualitative CLSM findings not only supported our quantitative results but also implied that the combination therapy is fungicidal rather than fungistatic (Figure 2B).

## 4. Discussion

Recurrent vulvovaginal candidiasis (RVVC) is a difficult-to-treat condition affecting many women of reproductive age. It clinically comprises an acute, symptomatic phase and a chronic, likely biofilm-associated phase that may be clinically asymptomatic [19,38]. The chronic phase of RVVC appears to be more challenging to manage as the current CDC-recommended antifungal therapy is unable to eradicate *Candida* biofilm cells [19]. We previously suggested that targeting *Candida* biofilms is crucial for the discovery of more effective antifungal therapies for this troublesome disease [19,20].

The current CDC guideline recommends using fluconazole for an extended period to treat RVVC [3]. In vitro studies by our group and many others have shown that *C. albicans* grown as abiotic or biotic biofilms does not respond to azole treatment [21,22,24,32,33]. We have gathered experimental and clinical evidence to support that *Candida* biofilm growth on the vaginal epithelium is the root cause of the persistence of RVVC after standard antifungal therapies [19,20]. We questioned whether the current treatment guideline could be further optimized and the use of CAM could be rationalized for the management of RVVC. Our hypothesis was that a higher anti-biofilm efficacy might be achieved when CDC-recommended conventional antifungals and clinically available CAM were combined.

With the introduction of novel antifungals oteseconazole and ibrexafungerp and their recent FDA approvals, clinical management of RVVC may be improving [12]. These novel antifungals, however, are costly and have very limited availability and global access. In the current study, we assessed the efficacy of conventional antifungals and CAM agents against *Candida* planktonic cells and in vitro biofilms, as single or combination therapies. While most of the selected agents retained activity against planktonic *Candida* cells, very few demonstrated measurable efficacy against biofilm-associated cells. The low anti-biofilm efficacy of selected antifungals may explain the suboptimal effectiveness of current treatment for RVVC in clinical settings. Another factor that may further confound the clinical effectiveness of antifungal therapies is the acidic microenvironment of the human vagina, which has been reported to affect the activity of many antimicrobials [32]. Routine antifungal susceptibility testing was carried out in microbiology diagnostic laboratories at a neutral pH between 7.0 and 7.4. Patients with fungal infections often present a vaginal pH which is weakly acidic (pH of 4.7 ± 0.8) [38]. The acidic vaginal pH may affect the efficacy of antifungals such as boric acid and clotrimazole and needs to be taken into consideration when developing a novel antifungal therapy for RVVC. In the current study, the echinocandin representative caspofungin is the best-performing single drug among the selected antifungals and effectively killed *Candida* biofilms in an acidic environment. Echinocandin, however, is not recommended for RVVC due to the low cost-effectiveness, requiring intravenous administration, and regulatory limitations. Fluconazole, clotrimazole, and allicin alone only showed limited activity against biofilms formed by *Candida* vaginal isolates. This is in line with the limited long-term clinical effectiveness of these drugs against RVVC [25,39]. Combining fluconazole at a concentration that can be achieved by oral administration and povidone-iodine at a concentration that can be reached and maintained by topical application may eradicate *Candida* biofilms to a level that is comparable to caspofungin. The combination of fluconazole and boric acid did not show significant superiority to fluconazole alone in our study.

Methodologically, we employed the XTT assay to screen anti-biofilm activities of single or combination antifungal therapies at pH = 7.2, followed by confirming the efficacy at a vaginal pH of 4.2 with a more accurate cell enumeration-based biofilm CFU assay and CLSM. The XTT screening assay was only carried out for biofilm susceptibility tests at a neutral pH, owing to the low sensitivity and accuracy of this assay under an acidic testing condition, as found in our preliminary study and also reported by others [40]. A low pH inhibits cell metabolism, destabilizes the XTT reagent, and reduces the intensity of the coloured formazan product in the XTT assay [40].

This study is limited by the absence of a biofilm-negative *Candida* strain and a fluconazole-resistant control strain, as well as by the relatively small number of clinical isolates included in the assessment. These clinical isolates have been used in our previous studies [19,20] and clinical and microbiological information specific to these strains has been published. The conclusion drawn from the current study may not be universally applicable to other *Candida* strains from patients with RVVC or VVC. Another limitation of this study is the in vitro nature. A large-scale, multi-centre clinical trial may be a practical measure that facilitates the translation of our in vitro findings to clinical application.

In conclusion, this study demonstrates the synergistic interactions between the CDC-recommended fluconazole and the CAM povidone-iodine in managing RVVC. Findings from this study may guide clinicians to choose antifungal regimens for the difficult-to-cure RVVC on a more scientific and evidence-based basis.

## Figures and Tables

**Table 1:** Susceptibility of conventional and CAM antifungals against *Candida* planktonic cells.

Strains	pH	Fluconazole (mg/L)		Caspofungin (mg/L)		Clotrimazole (mg/L)		Boric Acid (mg/L)		Povidone-Iodine (mg/L)		Galic Extract (mg/L)	
		MIC	MFC	MIC	MFC	MIC	MFC	MIC	MFC	MIC	MFC	MIC	MFC
SC5314	7.2	0.5	0.5	0.125	0.125	0.25	0.25	128	128	2048	2048	512	512
	4.2	0.125	0.5	0.125	0.125	4	4	1024	1024	1024	1024	512	512
	Log_2_ (fold change)	−2	0	0	0	4	4	3	3	−1	−1	0	0
VVC2	7.2	0.125	0.25	0.125	0.125	0.5	1	512	512	512	512	512	512
	4.2	0.25	0.25	0.125	0.125	4	4	2048	2048	1024	1024	512	512
	Log_2_ (fold change)	1	0	0	0	3	2	2	2	1	1	0	0
VVC4	7.2	1	1	0.125	0.125	1	1	512	512	2048	2048	512	512
	4.2	0.25	0.25	0.125	0.125	2	4	2048	2048	1024	1024	512	512
	Log_2_ (fold change)	−2	−2	0	0	1	2	2	2	−1	−1	0	0
QY4	7.2	1	1	0.125	0.125	1	1	512	512	2048	2048	512	512
	4.2	0.5	0.5	0.125	0.125	2	4	1024	1024	1024	1024	512	512
	Log_2_ (fold change)	−1	−1	0	0	1	2	1	1	−2	−2	0	0
QY3	7.2	0.5	0.5	0.125	0.125	0.5	1	1024	1024	2048	2048	512	512
	4.2	0.125	0.125	0.125	0.125	4	8	2048	2048	512	512	512	512
	Log_2_ (fold change)	−2	−2	0	0	3	3	1	1	−2	−2	0	0

**Table 2:** Interactions between fluconazole and CAM agents.

Strains	pH	MICs as Single Drug (mg/L)		MICs in Combination (mg/L)		FIC Index	Interpretation
		Fluconazole	Boric Acid	Fluconazole	Boric Acid		
SC5314	4.2	2	1024	1	512	1.00	Additive
	7.2	2	128	0.5	32	0.50	Synergistic
VVC2	4.2	0.5	2048	0.25	256	0.63	Additive
	7.2	0.5	512	0.25	32	0.56	Additive
VVC4	4.2	4	2048	2	1024	1.00	Additive
	7.2	2	512	2	256	1.50	Indifferent
QY4	4.2	2	1024	2	32	1.03	Indifferent
	7.2	4	512	1	256	0.75	Additive
QY3	4.2	2	2048	0.25	1024	0.63	Additive
	7.2	2	1024	1	256	0.75	Additive
		Fluconazole	Povidone-iodine	Fluconazole	Povidone-iodine		
SC5314	4.2	2	1024	0.125	512	0.56	Additive
	7.2	2	2048	1	64	0.53	Additive
VVC2	4.2	0.5	1024	0.25	32	0.53	Additive
	7.2	0.5	512	0.125	128	0.50	Synergistic
VVC4	4.2	4	1024	0.125	512	0.53	Additive
	7.2	2	2048	1	64	0.53	Additive
QY4	4.2	2	1024	0.25	256	0.38	Synergistic
	7.2	4	2048	1	128	0.31	Synergistic
QY3	4.2	2	512	0.25	128	0.38	Synergistic
	7.2	2	2048	1	32	0.52	Additive
		Fluconazole	Allicin	Fluconazole	Allicin		
SC5314	4.2	2	512	1	256	1.00	Additive
	7.2	2	512	2	128	1.25	Indifferent
VVC2	4.2	0.5	512	0.5	256	1.50	Indifferent
	7.2	0.5	512	0.125	64	0.38	Synergistic
VVC4	4.2	4	512	1	128	0.50	Synergistic
	7.2	2	512	2	64	1.13	Indifferent
QY4	4.2	2	512	0.25	128	0.38	Synergistic
	7.2	4	512	2	256	1.00	Indifferent
QY3	4.2	2	512	1	256	1.00	Indifferent
	7.2	2	512	0.5	256	0.75	Additive

**Table 3:** Susceptibility of conventional antifungals and CAM agents against *Candida* biofilms.

Strains	pH	BMIC_50_ of Single Antifungals (mg/L)						BMIC_50_ of CAMs in Combination with Fluconazole at 8 mg/L (mg/L)		
		Fluconazole	Clotrimazole	Caspofungin	Boric Acid	Povidone-Iodine	Allicin	Boric Acid	Povidone-Iodine	Allicin
SC5314	7.2	128	32	16	512	4096	1024	1024	1024	512
VVC2	7.2	>128	>128	4	4096	>4096	4096	2048	2048	4096
VVC4	7.2	64	64	4	2048	>4096	>4096	2048	2048	4096
QY4	7.2	>128	128	32	>4096	>4096	>4096	>4096	2048	>4096
QY3	7.2	64	64	32	>4096	4096	4096	>4096	2048	4096

## Data Availability

The original contributions presented in this study are included in the article. Further inquiries can be directed to the corresponding authors.
